# Selective Versus Non-Selective Thoracic Fusion for Adolescent Idiopathic Scoliosis Lenke 1B/1C Curves: Multi-Center Clinical and Radiographic Analysis at 2-Year Follow-Up

**DOI:** 10.3390/medicina61050909

**Published:** 2025-05-17

**Authors:** Yoshinari Miyaoka, Masashi Uehara, Tomohiro Banno, Shoji Seki, Tetsuro Ohba, Hiroki Oba, Shota Ikegami, Terue Hatakenaka, Daisuke Kurogochi, Takuma Fukuzawa, Tetsuhiko Mimura, Shinji Sasao, Hirotaka Haro, Yoshiharu Kawaguchi, Yukihiro Matsuyama, Jun Takahashi

**Affiliations:** 1 Department of Orthopaedic Surgery, Shinshu University School of Medicine, 3-1-1 Asahi, Matsumoto 390-8621, Nagano, Japan; miyauniv@shinshu-u.ac.jp (Y.M.);; 2 Department of Orthopaedic Surgery, Hamamatsu University School of Medicine, 1-20-1 Handayama, Higashi Ward, Hamamatsu 431-3192, Shizuoka, Japan; tomohiro.banno0311@gmail.com (T.B.);; 3 Department of Orthopaedic Surgery, Toyama University School of Medicine, 2630 Sugitani, Toyama 930-0194, Toyama, Japan; seki@med.u-toyama.ac.jp (S.S.);; 4 Department of Orthopaedic Surgery, Yamanashi University School of Medicine, 1110 Shimokato, Chuo 409-3898, Yamanashi, Japan; tooba@yamanashi.ac.jp (T.O.);

**Keywords:** selective thoracic fusion, adolescent idiopathic scoliosis, SRS-22r scores

## Abstract

*Background and Objectives*: This retrospective cohort study compared selective thoracic fusion (STF) and non-STF for adolescent idiopathic scoliosis (AIS) Lenke 1B/1C curves. Although STF is considered an attractive option for patients with a compensatory lumbar curve, direct clinical comparisons between STF and non-STF remain limited. *Materials and Methods*: AIS patients (≥2 years follow-up) undergoing posterior spinal fusion were divided into STF (57 cases) and non-STF (21 cases) groups. The correction rates of their main thoracic (MT) and thoracolumbar/lumbar (TL/L) curves, coronal balance, and SRS-22r scores were statistically compared. *Results*: Two years after the operation, while MT curve correction and coronal balance showed no significant differences, TL/L curve correction was significantly higher in the non-STF group. In contrast, the STF group had a significantly higher SRS-22r function score, with comparable results for self-image and satisfaction. *Conclusions*: Both STF and non-STF present distinct characteristics that should be considered to optimize surgical decision-making.

## 1. Introduction

Adolescent idiopathic scoliosis (AIS) typically develops between 10 and 18 years of age and results in progressive three-dimensional spinal deformities that frequently require surgical intervention. Since the 1990s, posterior spinal fusion with pedicle screw constructs has become the standard surgical approach for AIS, demonstrating favorable outcomes in terms of deformity correction and the maintenance of global spinal balance [1,2]. The surgical management of AIS remains a subject of debate, particularly in terms of the optimal extent of fixation. The Lenke 1B and 1C curve patterns are approached using two primary surgical strategies: selective thoracic fusion (STF) and non-selective thoracic fusion (non-STF) [3]. While STF aims to preserve lumbar mobility while correcting thoracic curvature, non-STF achieves greater overall spinal correction but may reduce lumbar function due to a longer fusion construct [4].

The STF approach to a primary thoracic curve with a compensatory lumbar curve was first advocated by Moe in 1958 [5] and is still considered the gold standard for treating this type of curve [6]. Good long-term clinical results and ranges of motion can be obtained by excluding the middle and lower lumbar spine from the STF fixation range [7,8]. Recent studies have demonstrated that spontaneous lumbar curve correction following STF is often sufficient, yet concerns regarding coronal decompensation and sagittal balance remain. Indeed, key clinical questions include whether STF causes a higher rate of postoperative coronal decompensation compared with non-STF and how these two surgical strategies differ in terms of functional outcomes and patient satisfaction. While STF has the potential to preserve lumbar motion and reduce surgical burden, its indication remains controversial, particularly in AIS patients with Lenke 1B or 1C curves and borderline modifiers. Moreover, few multi-center studies have compared the 2-year outcomes of STF and non-STF, limiting their broader clinical applicability. The absence of standardized criteria for selecting STF or non-STF has also contributed to the variability in surgical decision-making across institutions. Clarifying mid-term outcomes is thus essential for improving consistency in fusion level selection and guiding surgical planning in AIS.

This study hypothesized that STF could achieve superior postoperative coronal balance and functional outcomes in comparison to non-STF while maintaining comparable patient satisfaction. We conducted an observational cohort study to evaluate the radiographic and clinical outcomes of STF and non-STF two years post operation.

## 2. Materials and Methods

### 2.1. Study Design

This investigation retrospectively analyzed AIS patients with Lenke 1B or 1C curves recruited from four university hospitals. All patients received posterior spinal fusion using an all-pedicle screw construct and underwent a minimum 2-year follow-up.

To minimize institutional variability in the measurements, all radiographic and clinical assessments were standardized across participating centers. Radiographs were taken under standardized conditions (standing posteroanterior position, neutral pelvis, arms flexed at 90 degrees) to ensure consistency.

The sample size was determined based on a preliminary power analysis using the expected differences in SRS-22r scores and coronal balance measurements from previous reports. A minimum of 20 patients per group was estimated to be required to achieve 80% statistical power with a significance level of 0.05. Patients meeting the eligibility criteria were consecutively enrolled between 2009 and 2020.

### 2.2. Patient Selection

Patients were classified into the two groups based on their lowest instrumented vertebra (LIV). The STF group had their LIV at or above the apical vertebra of the thoracolumbar/lumbar (TL/L) curve. The non-STF group possessed a LIV below the apical vertebra of the TL/L curve. The exclusion criteria were as follows: patients who had previously undergone spinal correction surgery, age <21 years, Cobb angle ≥70° (i.e., <70° was excluded), and follow-up period <2 years.

### 2.3. Radiographic Evaluation

Standing whole-spine posteroanterior neutral-position radiographs were performed preoperatively and 2 years after surgery. Preoperative and postoperative radiographs were assessed for upper thoracic (UT), main thoracic (MT), and TL/L Cobb angles; coronal balance (C7 plumb line–central sacral vertical line [CSVL] distance); pelvic obliquity angle; clavicle angle (CA); and thoracic/thoracolumbar kyphosis. L4 and L3 tilt angles were also analyzed to evaluate lower lumbar compensation.

The flexibility of the TL/L curve was evaluated radiographically using standard preoperative side-bending views. A TL/L curve was considered flexible if it was corrected to <25° in accordance with the Lenke classification criteria [9].

We defined coronal decompensation as a change in coronal balance ≥2 cm, LIV coronal position shift ≥2 cm, thoracic trunk shift change ≥2 cm, or a LIV tilt angle change ≥10°. The pelvic obliquity angle was defined as the angle between a horizontal line and the line connecting the bilateral iliac crests in standing posteroanterior radiographs. These calculations were obtained using standard digital imaging software. All measurements were retrospectively performed by experienced clinicians who were blinded to surgical decision-making.

### 2.4. Clinical Assessment

Patient-reported outcomes were evaluated using the Scoliosis Research Society-22r (SRS-22r) questionnaire both preoperatively and 2 years after surgery.

### 2.5. Statistical Analysis

Inter-group comparisons were performed using Welch’s *t*-test or Fisher’s exact test, with significance set at *p* < 0.05. All statistical analyses were performed with EZR software Ver. 1.50 (Saitama Medical Center, Jichi Medical University, Saitama, Japan).

### 2.6. Ethical Considerations

This study was approved by the ethics review boards of the participating institutions (No. 5091, approved date: 29 March 2021). Informed consent was obtained from all patients and their guardians prior to study inclusion.

## 3. Results

We categorized 57 patients into the STF group (Lenke 1B: 34 cases; Lenke 1C: 23 cases) and 21 patients into the non-STF group (Lenke 1B: 3 cases; Lenke 1C: 18 cases), which had mean fused vertebrae numbers of 9.8 ± 1.5 and 11.7 ± 0.7, respectively (Table 1). A significant difference was noted between the STF and non-STF groups in terms of their preoperative TL/L curve magnitude (35.0° ± 7.9° vs. 41.2° ± 5.6°, *p* < 0.05) and L4 tilt (9.3° ± 4.0° vs. 12.9° ± 4.4°, *p* < 0.05) (Table 2).

### 3.1. Radiographic Outcomes

TL/L curve correction was significantly greater in the non-STF group than in the STF group two years after the operation (79% ± 14.2% vs. 58% ± 18.1%, *p* < 0.05). Although STF tended to achieve better coronal balance, this difference was not statistically significant (0.9 ± 0.7 cm vs. 1.1 ± 0.8 cm, *p* = 0.256). We observed no significant differences in UT curve correction, MT curve correction, or CA (Table 2). Both L4 and L3 tilt angles showed a significantly more improved alignment in the non-STF group (*p* < 0.05). The coronal decompensation rate two years post surgery was comparable between the STF and non-STF groups (8.8% vs. 9.5%, *p* = 0.78).

### 3.2. Clinical Outcomes

The STF group achieved a significantly higher SRS-22r function score than the non-STF group two years after the operation (4.8 ± 0.4 vs. 4.5 ± 0.4, *p* < 0.05), while similar scores were seen for self-image (3.9 ± 0.6, vs. 3.8 ± 0.7, *p* = 0.376) and satisfaction (4.1 ± 0.7 vs. 4.2 ± 0.4, *p* = 0.679) (Table 3). The mental health score was significantly higher in the non-STF group preoperatively (4.3 ± 0.6 vs. 3.9 ± 0.7, *p* < 0.05) and became balanced postoperatively (4.2 ± 0.6 vs. 4.3 ± 0.5, *p* = 0.491). No patients in the STF group required fusion extension due to postoperative coronal imbalance during the 2-year follow-up period.

A sub-analysis of the Lenke 1C curves revealed that the STF group experienced lower TL/L curve correction (49.5% ± 17.3% vs. 78.2% ± 15.0%, *p* < 0.05), although the SRS-22r self-image (3.9 ± 0.8, vs. 3.8 ± 0.7, *p* = 0.915) and satisfaction scores (4.0 ± 0.8, vs. 4.1 ± 0.7, *p* = 0.519) at the two-year follow-up were similar between the groups (Table 4).

## 4. Discussion

This study demonstrated that STF achieved comparable postoperative coronal balance and superior SRS-22r function scores to those of non-STF two years after the operation. In contrast, non-STF provided greater TL/L curve correction and better radiographic alignment in the lower lumbar segments. These findings suggest that while non-STF is effective at achieving radiographic goals, STF may offer a greater preservation of lumbar mobility and mid-term functional capacity, both critical considerations for long-term quality of life in AIS patients. Although the difference in TL/L correction is clinically relevant, the preservation of function may weigh more heavily when determining a surgical strategy. Importantly, the similarity in patient-reported self-image and satisfaction between groups indicates that aggressive radiographic correction may not necessarily translate into improved subjective outcomes. These findings underscore the complex balance between achieving optimal radiographic correction and preserving functional mobility in fusion level decisions for Lenke 1B/1C curves.

The primary goal of STF is to maintain overall coronal and sagittal alignment while allowing for the spontaneous correction of the unfused lumbar curve. Lenke et al. [10] established the AIS classification system and proposed criteria for STF in Lenke 1C curves, including a compensatory TL/L curve that corrects to the CSVL and bends to less than 25° on side-bending, with thoracolumbar kyphosis (T10–L2) of less than 20°. These criteria laid the foundation for selecting STF candidates and continue to serve as a reference for surgical planning. Since then, additional predictive factors—such as lumbar Cobb angle, coronal balance, and thoracolumbar flexibility—have been emphasized in the literature as enhancing patient selection strategies.

In 2014, Schulz et al. [11] proposed a triad of predictors for successful STF: a lumbar Cobb angle <26°, coronal balance within 2 cm, and deformity flexibility index <4. Patients who fulfilled these criteria experienced improved postoperative coronal alignment and better functional outcomes. Despite these advancements, no universally accepted guidelines currently exist, and the absence of a standardized algorithm likely contributes to ongoing variability in STF’s application and results. In our study, surgical decisions were often based on individual surgeons’ experience and institutional preferences, which may not have consistently reflected best practices. Although surgical strategies were determined according to widely accepted clinical criteria and surgeon discretion, the non-randomized nature of this retrospective study might have introduced selection bias. In particular, differences in baseline curve severity and flexibility could have influenced both radiographic and functional outcomes. These limitations should be taken into account when interpreting the comparative results of STF and non-STF. Nonetheless, the consistency of the radiographic and clinical trends across multiple centers supports the validity of the findings. As research advances, there is a growing need for evidence-based, individualized frameworks that can guide the selection of STF versus non-STF with greater reliability and consistency.

Our study found that coronal balance was similar the between STF and non-STF groups two years after surgery (0.9 cm ± 0.7 cm vs. 1.1 cm ± 0.8 cm, *p* = 0.26), with no significant differences in UT or MT curve correction. Non-STF achieved greater TL/L curve correction (79% ± 14.2% vs. 58% ± 18.1%) and more substantial L4 tilt reduction (7.1° ± 4.2° vs. 4.7° ± 3.7°, *p* < 0.05), demonstrating its efficacy in correcting lower spinal deformity. However, these radiographic benefits did not translate into superior patient-reported outcomes. Self-image and satisfaction scores were statistically comparable between the groups, while STF demonstrated significantly better function scores (4.8 ± 0.4 vs. 4.5 ± 0.4). Although function scores were significantly higher in the STF group, this difference did not reach the minimal clinically important difference (MCID), indicating limited clinical relevance. Nevertheless, these findings support the view that keeping lumbar motion segments unfused may contribute to better functional status and long-term biomechanical advantages [12]. The data also suggest that radiographic perfection does not always equate to improved patient perception or satisfaction in AIS surgery.

Despite these strengths, STF is not without its issues. Residual TL/L curvature and postoperative coronal decompensation remain important clinical issues. Prior studies have associated increased L4 tilt with lumbar pain and disc degeneration [13], although our cohort experienced minimal incidences of low back pain, even in patients with residual curves. This aligns with previous findings indicating that while L4 tilt may predict disc degeneration at skeletal maturity, its short-term clinical impact is limited [14,15]. Moreover, postoperative coronal decompensation has been linked to poor outcomes across multiple SRS-22r domains [3,15,16,17]. Although revision surgery is rarely necessary, an L3 tilt ≥16° has been identified as a risk factor for progressive curve deterioration over time [14]. In our study, the incidence of coronal decompensation was comparable between the STF and non-STF groups (8.8% vs. 9.5%), suggesting that, with appropriate selection, STF does not increase this risk. Looking ahead, long-term studies are essential to evaluate the durability of surgical results, disc health, and patient quality of life. Advanced technologies such as 3D imaging, machine learning-based prediction tools, and motion-preserving techniques like vertebral body tethering or hybrid constructs may further refine surgical strategies. While non-STF remains a valid approach for maximizing correction, STF continues to offer distinct advantages in the preservation of mobility. Individualized decision-making remains central to optimizing long-term outcomes in AIS.

Given the relatively young age of our cohort (average age: 14–15 years), the current results may not fully indicate long-term postoperative outcomes, particularly in terms of function and spinal balance. The real risk of curve progression, adjacent segment degeneration, and reduced lumbar mobility may become evident in later years. Therefore, extended follow-up is essential to confirm the durability and safety of each surgical approach.

## 5. Limitations

This retrospective multi-center study is subject to inherent selection bias along with the inconsistent availability of data. Variations in preoperative Cobb angles between Lenke 1B and 1C curves may have also influenced the results. Future studies with larger, standardized datasets and longer follow-up periods are needed to confirm our results and better understand the extended implications of STF and non-STF procedures.

## 6. Conclusions

STF achieved comparable postoperative coronal balance and better functional outcomes than non-STF, although it produced less TL/L curve correction. No cases of coronal decompensation were observed in the STF group at the 2-year follow-up, while self-image and satisfaction scores were similar between groups. The distinct advantages and disadvantages identified in this study may support surgical decision-making by highlighting the characteristics of each approach. Larger prospective studies with extended follow-up periods are warranted to validate these findings and create evidence-based guidelines for fusion level selection in AIS patients.

## Figures and Tables

**Table 1:** Patient demographics.

	STF Group	Non-STF Group	*p*-Value *
Characteristic	(*n* = 57)	(*n* = 21)	
Mean age (years)	15.0 ± 2.1	14.0 ± 1.6	
Sex			
Male	4	1	
Female	53	20	
Lenke classification			
Type 1B (*n* = 37)	34	3	
Type 1C (*n* = 41)	23	18	
Mean fused vertebrae	9.8 ± 1.5	11.7 ± 0.7	<0.05
Operative time (min)	231 ± 46.5	278 ± 25.4	<0.05
Operative blood loss (mL)	588 ± 366	521 ± 475	0.560

Data are expressed as a number or mean ± standard deviation. STF: selective thoracic fusion. * Determined by Welch’s *t*-test or Fisher’s exact test.

**Table 2:** Radiographic parameters before and after surgery.

	STF Group	Non-STF Group	*p*-Value *
Characteristic	(*n* = 57)	(*n* = 21)	
PT Cobb angle (°)			
Preop.	25.0 ± 7.7	21.8 ± 7.2	0.096
Postop. 2Y	13.0 ± 7.3	12.7 ±5.8	0.835
MT Cobb angle (°)			
Preop.	49.0 ± 7.9	49.9 ± 4.7	0.554
Postop. 2Y	16.9 ± 9.1	14.2 ± 7.2	0.182
TL/L Cobb angle (°)			
Preop.	35.0 ± 7.9	41.2 ± 5.6	<0.05
Postop. 2Y	15.0 ± 8.0	8.5 ± 5.8	<0.05
Coronal balance			
C7PL-CSVL (cm)			
Preop.	1.2 ± 0.8	1.7 ± 1.0	<0.05
Postop. 2Y	0.9 ± 0.7	1.1 ± 0.8	0.256
Pelvic obliquity angle (°)			
Preop.	0.8 ± 1.3	0 ± 1.4	<0.05
Postop. 2Y	0.2 ± 1.6	−0.5 ± 1.6	0.100
CA (°)			
Preop.	3.1 ± 2.2	2.8 ± 2.4	0.523
Postop. 2Y	2.3 ± 1.7	2.0 ± 1.7	0.452
L4 tilt (°)	-	-	
Preop.	9.3 ± 4.0	12.9 ± 4.4	<0.05
Postop. 2Y	7.1 ± 4.2	4.7 ± 3.7	<0.05
L3 tilt (°)			
Preop.	4.2 ± 5.3	8.4 ± 8.4	<0.05
Postop. 2Y	4.2 ± 4.9	0.8 ± 3.8	<0.05
Sagittal balance			
T5-T12 kyphosis (°)	-	-	
Preop.	13.8 ± 8.0	18.8 ± 12.3	0.100
Postop. 2Y	22.0 ± 9.2	21.0 ± 7.9	0.668
T10-L2 kyphosis (°)			
Preop.	2.7 ± 9.4	5.6 ± 8.2	0.195
Postop. 2Y	5.8 ± 7.3	5.9 ± 5.8	0.938
T12-S1 lordosis (°)			
Preop.	51.3 ± 11.9	46.0 ± 13.6	0.118
Postop. 2Y	46.0 ± 13.5	49.0 ± 14.2	0.330

All data are expressed as a mean ± standard deviation. STF: selective thoracic fusion; C7PL: C7 plumb line; CSVL: central sacral vertical line; CA: clavicle angle. * Determined by Welch’s *t*-test or Fisher’s exact test.

**Table 3:** Comparisons of SRS-22r scores before and after surgery.

	STF Group	Non-STF Group	*p*-Value *
	(*n* = 57)	(*n* = 21)	
Preoperative			
Function	4.5 ± 0.4	4.5 ± 0.7	0.816
Pain	4.2 ± 0.6	4.4 ± 0.7	0.360
Self-image	3.0 ± 0.6	2.8 ± 0.5	0.119
Mental health	3.9 ± 0.7	4.3 ± 0.6	<0.05
Subtotal	3.9 ± 0.3	4.0 ± 0.4	0.511
2 years post operation			
Function	4.8 ± 0.4	4.5 ± 0.4	<0.05
Pain	4.5 ± 0.5	4.5 ± 0.7	0.687
Self-image	3.9 ± 0.6	3.8 ± 0.7	0.376
Mental health	4.3 ± 0.5	4.2 ± 0.6	0.491
Subtotal	4.4 ± 0.3	4.2 ± 0.5	0.212
Satisfaction	4.1 ± 0.7	4.2 ± 0.4	0.679
Total	4.4 ± 0.3	4.3 ± 0.4	0.308

All data are expressed as a mean ± standard error. SRS-22r: Scoliosis Research Society 22r questionnaire; STF: selective thoracic fusion. * Determined by Welch’s *t*-test.

**Table 4:** Comparisons of SRS-22r Scores in Lenke 1C patients before and after surgery.

	STF Group	Non-STF Group	*p*-Value *
	(*n* = 23)	(*n* = 18)	
Preoperative			
Function	4.7 ± 0.3	4.5 ± 0.7	0.919
Pain	4.3 ± 0.5	4.5 ± 0.6	0.126
Self-image	3.1 ± 0.5	2.9 ± 0.5	0.100
Mental health	3.9 ± 0.6	4.3 ± 0.5	<0.05
Subtotal	4.0 ± 0.3	4.0 ± 0.4	0.183
2 years post operation			
Function	4.7 ± 0.5	4.6 ± 0.4	0.165
Pain	4.5 ± 0.5	4.5 ± 0.6	0.608
Self-image	3.9 ± 0.8	3.8 ± 0.7	0.915
Mental health	4.4 ± 0.5	4.2 ± 0.7	0.397
Subtotal	4.4 ± 0.4	4.3 ± 0.4	0.397
Satisfaction	4.0 ± 0.8	4.1 ± 0.7	0.519
Total	4.4 ± 0.4	4.3 ± 0.4	0.600

All data are expressed as a mean ± standard error. SRS-22r: Scoliosis Research Society 22r questionnaire; STF: selective thoracic fusion. * Determined by Welch’s *t*-test.

## Data Availability

The authors confirm that the data supporting the findings of this study are available within the article or can be made available upon reasonable request.
